# The usefulness of tranexamic acid for bleeding symptoms of chronic consumptive coagulopathy complicated by aortic disease: a single-institute, retrospective study of 14 patients

**DOI:** 10.1186/s12959-022-00429-4

**Published:** 2023-01-25

**Authors:** Naruko Suzuki, Nobuaki Suzuki, Yuka Kawaguchi, Shuichi Okamoto, Takeshi Kanematsu, Akira Katsumi, Atsuo Suzuki, Shogo Tamura, Tetsuhito Kojima, Hitoshi Kiyoi, Tadashi Matsushita

**Affiliations:** 1 Department of Hematology and Oncology, Nagoya University Graduate School of Medicine, Nagoya, Japan; 2 Department of Transfusion Medicine, Nagoya University Hospital, Nagoya, Japan; 3 Department of Clinical Laboratory, Nagoya University Hospital, Nagoya, Japan; 4 Department of Hematology, National Center of Geriatrics and Gerontology, Obu, Japan; 5 Department of Medical Technique, Nagoya University Hospital, Nagoya, Japan; 6 Department of Integrated Health Sciences, Nagoya University Graduate School of Medicine, Nagoya, Japan; 7 Present address: Department of Medical Laboratory Science, Hokkaido University Graduate School of Health Science, Sapporo, Japan; 8 Aichi Health Promotion Foundation, Nagoya, Japan

**Keywords:** Disseminated intravascular coagulation, Tranexamic acid, Aortic aneurysm, Aneurysm, Dissecting, Endovascular procedures

## Abstract

**Background:**

Tranexamic acid (TXA) is an antifibrinolytic drug that blocks lysine-binding sites on the profibrinolytic enzyme plasminogen. Aortic diseases with chronic consumption coagulopathy may lead to disseminated intravascular coagulation (DIC) and cause fatal bleeding. Although the use of antifibrinolytic agents in DIC is generally not recommended due to enhanced fibrin deposition risking thrombotic symptoms, the efficacy of TXA has been reported in several cases of DIC with aortic diseases. However, the efficacy and safety of TXA for bleeding symptoms of chronic consumption coagulopathy with aortic diseases have not been studied in detail.

**Methods:**

We evaluated the efficacy of TXA in 14 patients with chronic consumptive coagulopathy due to aortic disease complicated by bleeding symptoms. Changes in coagulation and fibrinolysis parameters from baseline were analyzed with Wilcoxon matched-pairs signed-rank tests, excluding missing values. Kaplan-Meier curves were used to analyze overall survival.

**Results:**

Median age was 78.5 years (range, 66–89 years) and median observation period was 448 days (range, 0–2282 days). Twelve patients had chronic renal failure and 1 patient had chronic liver failure. Before starting treatment, median Japanese Ministry of Health and Welfare DIC diagnostic criteria score was 8 (range, 4–11) and median platelet count was 64 × 10^9^/L (range, 25–97 × 10^9^/L). Twelve patients underwent evaluation of bleeding symptoms after introduction of TXA, and 10 of those 12 patients showed improved bleeding tendencies within 30 days (median, 5.0 days). One patient with chronic liver failure showed worsening of bleeding symptoms. Although only one patient was initiated TXA in combination with anticoagulants, no significant worsening of thrombotic events was observed within 30 days.

**Conclusions:**

TXA therapy appears effective against chronic consumptive coagulopathy with bleeding due to aortic disease, with few side effects.

**Supplementary Information:**

The online version contains supplementary material available at 10.1186/s12959-022-00429-4.

## Background

As Japan has a rapidly aging population and the numbers of patients with aortic disease are increasing year by year, the treatment demand for frail patients with aortic disease is expected to increase [1]. Aortic diseases such as aortic aneurysms and aortic dissection are known to be complicated by consumptive coagulopathy. Some cases lead to the hyperfibrinolytic form of disseminated intravascular coagulation (DIC), a condition in which various underlying diseases activate the coagulation system. Zhang et al. reported that the clinical courses in 22.1% of patients with aortic aneurysm were complicated by chronic consumptive coagulopathy, and 4% progressed to DIC, which can result in fatal bleeding complications [2, 3].

The optimal method for treating chronic consumptive coagulopathy is treatment of the primary disease [3]. However, in cases where the primary disease is difficult to control, anticoagulants and antifibrinolytics may be used to improve the symptoms of chronic consumptive coagulopathy-related thrombosis and bleeding [2].

Tranexamic acid (TXA) is an antifibrinolytic drug that blocks lysine-binding sites on plasminogen molecules. TXA shows hemostatic effects in conditions with bleeding tendencies [4]. As a result, this agent is used in certain conditions with abnormal bleeding or bleeding tendencies in which local or systemic hyperfibrinolysis is considered to be involved [5]. Prolonged TXA administration has been successfully used to treat bleeding in several cases of chronic DIC associated with aortic disease (CDAAD) [6–11]. However, no studies appear to have examined TXA for bleeding symptoms of chronic consumption coagulopathy with aortic diseases. We report a single-center, retrospective study of 14 cases in which TXA was used to treat consumption coagulopathy due to aortic disease complicated by bleeding symptoms.

## Methods

### Eligible patients

**Fig. 1:**
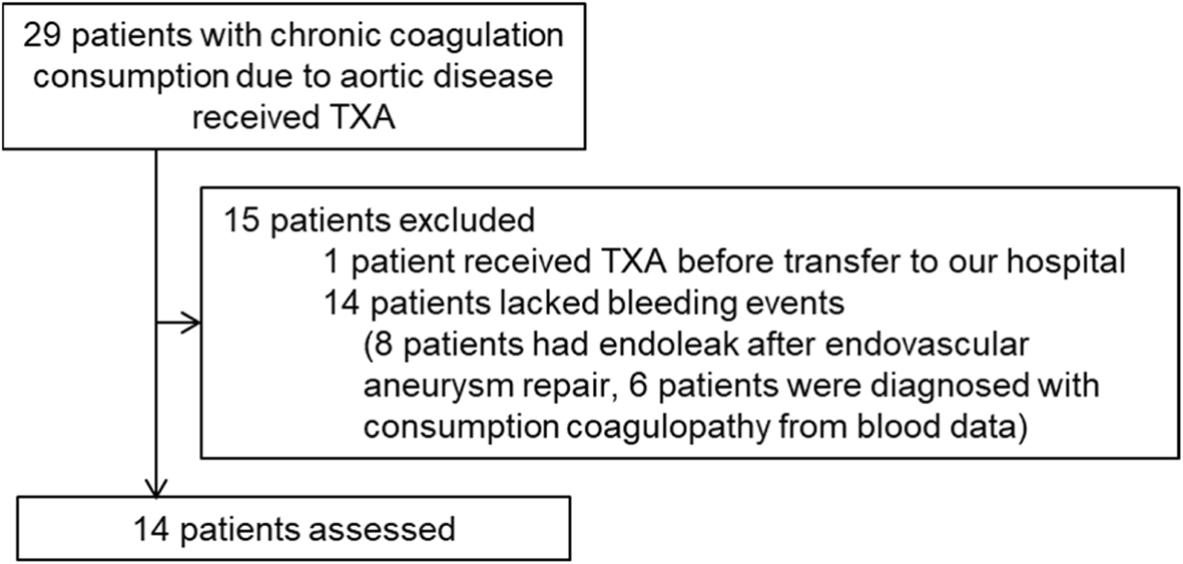
Patient selection. TXA: tranexamic acid

Patients with underlying aortic disease and a history of TXA administration at our hospital between January 2015 and July 2021 were registered, as shown in Fig. 1. Aortic disease was defined as a disorder of the aorta such as aortic aneurysm, aortic dissection, and aortic valve disease. One patient had received TXA from another hospital before transfer to our institution. Fourteen patients were administered TXA without bleeding symptoms. Among those 14 patients, six had no bleeding and were administered TXA monotherapy because of a gradual worsening in coagulation markers such as elevation of D-dimer. Eight patients received TXA for endoleak after endovascular aneurysm repair (EVAR). We evaluated improvements in bleeding symptoms with TXA in 14 patients. DIC score, bleeding symptoms, thrombotic complications, laboratory values, treatments, and outcomes were assessed. The change from baseline was analyzed with Wilcoxon matched pairs signed-rank test, excluding missing values. Kaplan-Meier curves were used to analyze overall survival. This study was performed in accordance with the Declaration of Helsinki and was approved by our ethics committee (approval no. 2022–0060).

### Criteria for diagnosis of DIC

The diagnostic criteria for DIC established by the Japanese Ministry of Health and Welfare (JMHW) were used to diagnose DIC [12]. The JMHW diagnostic criteria for DIC are shown in Supplemental Table 1 (see Additional file 1). Underlying disease, clinical symptoms, platelet count, fibrin degradation product (FDP), fibrinogen, and prothrombin time (PT) ratio were evaluated. JMHW DIC scores > 6 were considered diagnostic of DIC. A patient with a score of 6 was diagnosed as having DIC if at least two of the “Supplemental diagnostic laboratory results and findings” were met. Three points were deducted for complications of liver failure. Bleeding symptoms were defined in accordance with International Society on Thrombosis and Haemostasis bleeding criteria [13, 14]. Resolution of bleeding symptoms was assessed within 30 days after TXA administration.

Supplementary Table 1 DIC diagnostic criteria. DIC, disseminated intravascular coagulation; N.A. not applicable; PT, prothrombin time; FDP, fibrin and fibrinogen degradation products; TAT, thrombin anti-thrombin complex. Values of D-dimer can be converted to values of FDP

## Results

### Patient characteristics

The background characteristics of patients are shown in Table 1. Median age was 78.5 years (range, 66–89 years), and 11 patients were male. Prior to TXA administration, 9 patients had undergone EVAR, 7 had undergone open aortic aneurysm repair, and 1 had undergone transcatheter aortic valve implantation. Only one patient had no previous treatment for primary aortic disease. Median JMHW diagnostic criteria score for DIC was 8.0 (range, 4–11). Except for the 1 patient with liver failure, the 13 remaining patients (92.8%) met the diagnostic criteria for DIC. Bleeding symptoms were bloody sputum in 2 cases, subcutaneous hemorrhage in 2 cases, and difficulty achieving hemostasis after bleeding procedures in 10 cases. The procedures that caused difficulty in hemostasis were hemodialysis in 5 cases, cardiac catheterization in 1 case, central venous catheter insertion in 1 case, surgery in 2 cases, blood testing in 1 case, thoracic drainage in 1 case, and tooth extraction in 1 case. Two cases received more than 2 units of red cell transfusion, meeting the criteria for major bleeding. Bleeding events in other cases were clinically relevant non-major bleeding.

**Table 1:** Baseline characteristics

	*n* = 14	
Age, years, median (range, mean)	78.5	(66–89, 78.2)
Sex, male, n (%)	11	(78.5)
CKD (eGFR < 60), n (%)	12	(85.7)
Hemodialysis^a^, n (%)	6	(42.8)
Liver failure^b^, n	1	
JMHW DIC score, median (range, mean)	8.0	(4–11, 7.7)
Platelet count (×10^9^/L), median (range, normal range)	64	(25–97, 158–348)
FDP (μg/mL), median (range, normal range)	100	(19–270, < 10)
D-dimer (μg/mL), median (range, normal range)	44.3	(13.4–93.7, < 1.0)
FDP/D-dimer, median (range)	2.3	(1.2–4.2)
Fibrinogen (g/L), median (range, normal range)	1.4	(0.56–2.94, 2.0–4.0)
PT ratio, median (range)	1.15	(1.02–1.55)
ATIII (%), median (range, normal range)	78	(56–101, 80–120)
TAT (ng/mL), median (range, normal range)	45.9	(10.9–97.5, 0–4.0)
PIC (μg/mL), median (range, normal range)	5.0	(2.1–10.9, 0–0.8)
Bleeding event (overlap included), n	14	
Difficult hemostasis	10	
after hemodialysis	5	
after cardiac catheterization	1	
after central venous catheter insertion	1	
during or after surgery	2	
after blood test	1	
after thoracic drainage	1	
after tooth extraction	1	
Subcutaneous hemorrhage	2	
Bloody sputum	2	
Aortic treatment prior to TXA, n	13	
Number of aortic treatments prior to TXA, median (range)	2.0	(0–4)
EVAR, n	9	
Days from the most recent treatment, median (range)	1192	(4–5410)
Open aortic aneurysm repair, n	7	
Days from the most recent treatment, median (range)	678	(3-3088)
Transcatheter aortic valve implantation, n	1	
Days from the most recent treatment	88	
Transfusion 30 days prior to TXA, n	4	
RBC	4	
FFP	3	
Fibrinogen	2	
PC	2	

^a^ Includes cases in which planned hemodialysis was introduced the day after the start of TXA administration

^b ^Liver failure was defined as chronic liver disease with Child-Pugh B or C

*CKD* Chronic kidney disease, *eGFR* Estimated glomerular filtration rate, *JMHW* Japanese Ministry of Health and Welfare, *DIC* Disseminated intravascular coagulation, *FDP* Fibrin and fibrinogen degradation products, *PT* Prothrombin time, *CKD* Chronic kidney disease, *AT* Antithrombin, *TAT* Thrombin anti-thrombin complex, *PIC* Plasmin-α2 plasmin inhibitor complex, *TXA* Tranexamic acid, *EVAR* Endovascular aneurysm repair, *RBC* Red blood cell, *FFP* Fresh frozen plasma, *PC* Platelet concentrate

Twelve patients had chronic kidney disease (CKD) due to nephrosclerosis in 4 cases, cholesterol embolization in 1, impaired blood flow due to thrombus in 1, bilateral renal artery embolization by EVAR for endoleak in 1, right nephrectomy in 1, and unknown cause in 4. One patient had chronic liver injury caused by congested liver due to chronic heart failure and chronic renal failure.

Median platelet count was 64 × 10^9^/L (range, 25–97 × 10^9^/L). Median fibrinogen was 1.4 g/L (range, 0.56–2.94 g/L). Most cases displayed a near-normal PT ratio.

### Dosing and other treatments

Dosing of TXA and descriptions of transfusion, antiplatelet, and anticoagulation treatments are shown in Table 2 and Supplemental Table 2 (see Additional file 2). Thirteen of the 14 cases were evaluable. Median initial TXA dose was 4.5 mg/kg/day (range, 0.8–24.0 mg/kg/day). One patient with an initial dose of 1000 mg was reduced to 50 mg/day on day 3. In that case, hemodialysis had been introduced before initial TXA treatment. All patients continued low-dose TXA after hemostasis, in expectation of maintaining good control of consumptive coagulation. Median duration of treatment was 597 days (range, 6–2282 days). Six patients required platelet concentrate (PC) or plasma derivative transfusions within 30 days post-TXA. Two patients received transfusions of both fresh frozen plasma (FFP) and PC. Only 4 cases received one transfusion and no patients received regular transfusions. Six patients received antiplatelet or anticoagulant treatments before and after TXA. Two patients received short-term thrombomodulin before or after TXA for preoperative DIC treatment. No other cases met indications for antiplatelet or anticoagulant therapy for DIC treatment.

**Table 2:** Description of TXA and transfusion treatment

	*n* = 14	
TXA duration, days, median (range, mean)	597	(6–2282, 776)
Initial TXA dose, mg/day, median (range, mean)	250	(50–1000, 314)
Final TXA dose, mg/kg/day, median (range, mean)	5.5	(2.3–15.0, 5.8)
Final TXA dose, mg/day, median (range, mean)	250	(150–500, 282)
PC or plasma derivative transfusion within 30 days post-TXA, n	6	
FFP	4	
Fibrinogen	1	
PC	3	

*TXA* Tranexamic acid, *PC* Platelet concentrate, *FFP* Fresh frozen plasma

### Bleeding outcomes

Bleeding outcomes are shown in Table 3. The median observation period was 448 days (0–2282 days; mean, 744 days), with resolution of bleeding symptoms confirmed by 30 days in 10 cases. In the remaining 4 cases without resolution of bleeding symptoms within 30 days, 1 case experienced worsening bleeding, 1 case showed improvement of bleeding on day 33, and data were lacking in 2 cases. Of the 10 cases showing resolution of bleeding symptoms by 30 days, median time to improvement was 5.0 days (range, 2–22 days; mean, 6.2 days). Among the 11 cases that achieved a decrease in bleeding symptoms, TXA was continued for a median of 748 days (range, 21–2277 days; mean, 906 days) for bleeding prophylaxis. During the 30 days in which bleeding symptoms were observed, three cases received transfusion of 2 units of red blood cells (RBC), and one case with complications of sepsis on day 17 received transfusion of 8 units of RBCs. Although bleeding symptoms were under control, this patient died of sepsis on day 27.

**Table 3:** Bleeding outcomes and causes of death

	*n* = 14	
Decrease of bleeding symptoms, n (%)	11	(78.5)
Increase of bleeding symptoms, n	1	
No data, n	2	
Time to improvement within 30 days, days, median (range)	5.0	(2–22)
RBC transfusion, n	4	
Seizures, n	0	
Thrombosis, n	0	
Death, n (%)	5	(35.7)
Brain hemorrhage	1	
Infection	1	
Hemodialysis termination	1	
Chronic DIC	1	
Unknown	1	

*RBC* Red blood cell, *DIC* Disseminated intravascular coagulation

Median overall survival was 2119 days (mean, 1459 days). Five patients died, one each from discontinuation of hemodialysis, aspiration pneumonia, brainstem hemorrhage, chronic DIC, and unknown cause. In the patient who died of brainstem hemorrhage, TXA was administered for 30 months then discontinued due to gradual decline of renal function, and no association with TXA could be confirmed.

### Laboratory data

**Fig. 2:**
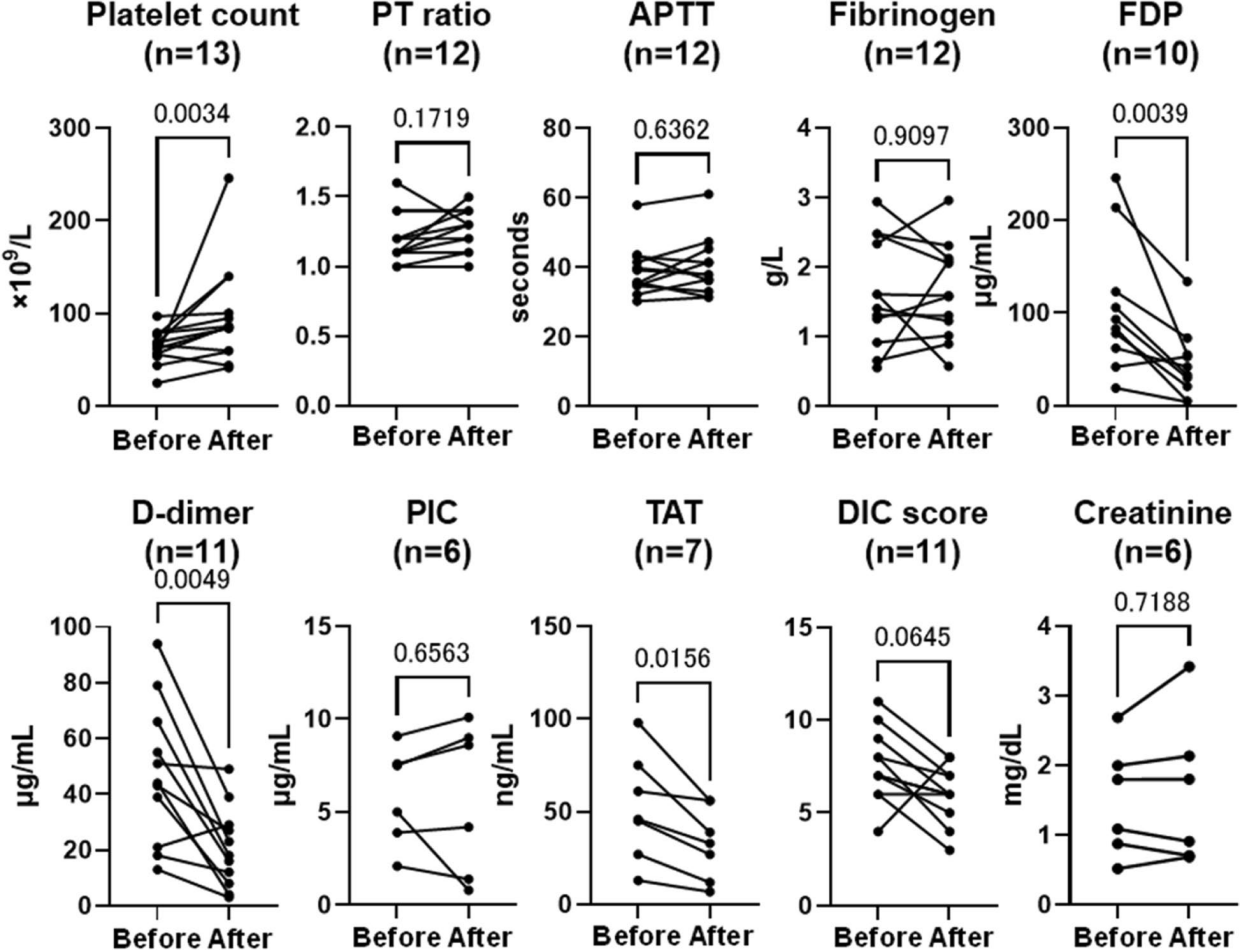
Changes in laboratory data before and after TXA initiation. Median laboratory data from day 2 to day 30 was analyzed as data after TXA initiation. DIC score was assessed using JMHW diagnostic criteria. TXA: tranexamic acid; DIC: disseminated intravascular coagulation; JMWH: Japanese Ministry of Health and Welfare; PT: prothrombin time; APTT: activated partial thromboplastin time; FDP: fibrin degradation product; PIC: plasmin-α2 plasmin inhibitor complex; TAT: thrombin-antithrombin complex

Comparing median values before and after initiation of TXA, significant improvements were seen in platelet count, FDP, D-dimer, and thrombin-antithrombin complex (TAT) (*p* < 0.05). No significant improvements were seen for PT ratio, activated partial thromboplastin time (APTT), fibrinogen, antithrombin, plasmin-alpha2 plasmin inhibitor (α2PI) complex (PIC), or JMWH DIC score (Fig. 2).

### Adverse events

None of the patients showed thrombosis or seizures as an adverse event (Table 3). Figure 2 shows changes in serum creatinine before and after TXA among non-dialysis patients. No significant increase in creatinine was evident after TXA administration (*p* = 0.7188).

## Discussion

Consumptive coagulopathy associated with aortic disease progresses to the state of enhanced fibrinolytic-type DIC (EFDIC). Yamada et al. reported that EFDIC is characterized by decreased platelet count, fibrinogen and α2PI and increased TAT, PIC, FDP and D-dimer, while PT and APTT are less prolonged [15]. Elevated PIC is also characteristic, particularly in EFDIC, with some previous reports describing PIC > 10 g/mL as characteristic of EFDIC. The data from this study showed a trend generally consistent with these characteristics of EFDIC, but median PIC was 5.0 g/mL (range, 2.1–10.9 μg/mL), which is low for EFDIC. Previous case reports of CDAAD showed PICs ranging from 0.8 to 10.6 μg/mL, generally similar to the present study [6–11, 16, 17]. The degree of PIC elevation in CDAAD may thus be characterized as “not elevated”.

The dose of TXA administered in this study was quite low, around 30% of the dose normally used, and resulted in a significant reduction in FDP, one measure of increased fibrinolysis, but little reduction in PIC. A possible reason explaining why such low doses were effective in improving bleeding symptoms is that fibrinolysis is not as severe in CDAAD and is not the only major cause of bleeding symptoms.

The abnormal coagulation seen in the present study was not only fibrinolytic, but also coagulopathic, considering that TAT was also elevated. Therefore, a complex coagulation abnormality was considered to be caused by an imbalance between coagulation and fibrinolysis, rather than a state of increased fibrinolysis alone, suggesting that the inhibition of fibrinolysis by TXA does not contribute to the improvement of bleeding symptoms alone. Another possibility is that TXA-induced fibrinolysis inhibition may lead to an improvement in overall coagulation balance, in turn leading to improved bleeding symptoms.

Although oral TXA produced improvements in bleeding tendency, no significant difference in JMHW DIC score was identified. This is because platelet count, fibrinogen, and FDP must improve significantly to be reflected in an improved score. Another reason may be that this scoring system reflects improvements not only in bleeding symptoms, but also in items not directly related to coagulability, such as underlying disease and organ damage. In any case, the effect of low-dose TXA appeared to be an improvement in bleeding tendency, while DIC may not be improved.

However, although not evaluated in the present study, Aoki et al. reported that concomitant use of long-term TXA (1500 mg/day for 6 months) in endovascular repair procedures resulted in a size reduction of aneurysms without any severe adverse events [18]. As such, TXA may not only directly improve blood coagulability, but also have an effect on the underlying aortic lesion, which would make this a very attractive treatment for CDAAD.

With regard to safety, deaths due to thrombotic complications have been reported with the use of TXA as a single agent in DIC with underlying diseases other than aortic disease [15, 19, 20]. However, no reports to date have described thrombosis with TXA monotherapy for CDAAD, and no thrombotic events occurred in the present study. No other side effects could be clearly attributed to TXA.

Retrospectively, all cases in the present study continued TXA for bleeding prophylaxis and CDAAD control after achieving hemostasis. This is because relapse of severe bleeding symptoms after TXA discontinuation have been reported, and prolonged concomitant use of anticoagulants and TXA has been reported as useful for controlling bleeding symptoms and DIC [10, 21]. However, heparin is mainly used as the anticoagulant, and injections cannot be avoided. Several reports have described direct oral anticoagulants as an effective additional oral pharmacotherapy for CDAAD [22, 23]. However, direct oral anticoagulants cannot be used in patients with reduced renal function and are therefore often unsuitable in patients with CDAAD, many of whom have CKD. TXA also requires dose adjustment in CKD patients, with Ma et al. recommending oral doses of 7.5–15 mg/kg/day for non-dialysis CKD patients [24]. The dose of approximately 5 mg/kg/day adopted in the present study can be administered to non-dialysis CKD patients without problems.

Finally, several limitations need to be considered in this study. First, this was a retrospective study of a small number of patients and TXA cannot be concluded to be effective based on improved outcomes. Two patients underwent EVAR during the observation period, and hemostatic effects of factors other than TXA cannot be ruled out. Future studies should assess the clinical efficacy of TXA in well-conditioned, prospective trials, while detailed assessment of blood coagulation performance is needed to determine exactly which coagulation findings are altered and which bleeding trends are improved by TXA.

## Conclusions

For bleeding symptoms due to consumptive coagulopathy associated with aortic disease, oral treatment with low-dose TXA resulted in improvement of bleeding symptoms without apparent side effects. Further studies are needed to validate this treatment.

## Supplementary Information

**Additional file 1: Supplemental Table 1.** DIC diagnostic criteria established by JMHW. Values of D-dimer can be converted to values of FDP. DIC: disseminated intravascular coagulation; JMHW: Japanese Ministry of Health and Welfare; PT: prothrombin time; FDP: fibrin and fibrinogen degradation products; TAT, thrombin-anti-thrombin complex; PIC: plasmin-α2 plasmin inhibitor complex.

**Additional file 2: Supplemental Table 2.** Descriptions of patients using antiplatelet or anticoagulant treatments before and after TXA. † Days before and after TXA (starting from the date of TXA initiation). TXA: tranexamic acid; NA: not applicable; DIC: disseminated intravascular coagulation.

## Data Availability

The datasets generated and analyzed during the present study are available from the corresponding author on reasonable request.

## Abbreviations

DIC: Disseminated intravascular coagulation
TXA: Tranexamic acid
CDAAD: Chronic DIC associated with aortic disease
EVAR: Endovascular aneurysm repair
JMHW: Japanese Ministry of Health and Welfare
FDP: Fibrin degradation product
PT: Prothrombin time
CKD: Chronic kidney disease
PC: Platelet concentrate
FFP: Fresh frozen plasma
RBC: Red blood cell
TAT: Thrombin-antithrombin complex
APTT: Activated partial thromboplastin time
α2PI: α2 plasmin inhibitor
PIC: Plasmin-α2 plasmin inhibitor complex
EFDIC: Enhanced fibrinolytic-type DIC
