# Supplementary material for: The usefulness of tranexamic acid for bleeding symptoms of chronic consumptive coagulopathy complicated by aortic disease: a single-institute, retrospective study of 14 patients
Source: Thromb J. 2023 Jan 25;21:10. doi: 10.1186/s12959-022-00429-4 (PMC9878879; doi:10.1186/s12959-022-00429-4)
Supplement: Supplementary file 1 — Additional file 1: Supplemental Table 1. DIC diagnostic criteria established by JMHW. Values of D-dimer can be converted to values of FDP. DIC: disseminated intravascular coagulation; JMHW: Japanese Ministry of Health and Welfare; PT: prothrombin time; FDP: fibrin and fibrinogen degradation products; TAT, thrombin-anti-thrombin complex; PIC: plasmin-α2 plasmin inhibitor complex. [file 12959_2022_429_MOESM1_ESM.docx]

Supplemental table 1

|  |  | Score |
| --- | --- | --- |
| Underlying disease | Absent | 0 |
|  | Present | 1 |
| Bleeding symptoms | Absent | 0 |
|  | Present | 1 |
| Organ dysfunction | Absent | 0 |
|  | Present | 1 |
| Platelet count (×10^9^/L) | >120 | 0 |
|  | > 80 and ≤120 | 1 |
|  | > 50 and ≤ 80 | 2 |
|  | ≤ 50 | 3 |
| PT ratio | < 1.25 | 0 |
|  | ≥ 1.25 and < 1.67 | 1 |
|  | ≥ 1.67 | 2 |
| Fibrinogen (g/L) | >1.5 | 0 |
|  | > 1.0 and ≤1.5 | 1 |
|  | ≤ 1.0 | 2 |
| FDP (μg/mL) | < 10 | 0 |
|  | ≥ 10 and < 20 | 1 |
|  | ≥ 20 and < 40 | 2 |
|  | ≥ 40 | 3 |
| Liver failure, hepatic cirrhosis, or severe hepatitis | Absent | 0 |
|  | Present | -3 |
| Supplemental diagnostic laboratory results and findings  1) Positive soluble fibrin monomer  2) Elevated D-dimer  3) Elevated TAT  4) Elevated PIC level  5) Emergence of an increasing trend in scores as the disease progresses.  In particular, a sharp decrease in platelet count or fibrinogen or a sharp increase in FDP within a few days.  6) Improvement with anticoagulation therapy. | | |
