# Supplementary material for: The usefulness of tranexamic acid for bleeding symptoms of chronic consumptive coagulopathy complicated by aortic disease: a single-institute, retrospective study of 14 patients
Source: Thromb J. 2023 Jan 25;21:10. doi: 10.1186/s12959-022-00429-4 (PMC9878879; doi:10.1186/s12959-022-00429-4)
Supplement: Supplementary file 2 — Additional file 2: Supplemental Table 2. Descriptions of patients using antiplatelet or anticoagulant treatments before and after TXA. † Days before and after TXA (starting from the date of TXA initiation). TXA: tranexamic acid; NA: not applicable; DIC: disseminated intravascular coagulation. [file 12959_2022_429_MOESM2_ESM.docx]

| **Case** | **Indication for treatment** | **Treatment** | **Initiation^†^** | **Bleeding^†^** | **Hemostasis^†^** | **Discontinuation^†^** | **Reason of Discontinuation** |
| --- | --- | --- | --- | --- | --- | --- | --- |
| **1** | Atrial fibrillation | Heparin, warfarin | –29 | 0 | 4 | 14 | Cessation of arrythmia |
| **2** | Atrial fibrillation | Rivaroxaban | –378 | –4 | 5 | –4 | Operation |
| **3** | Angina | Aspirin | –670 | –2 | 2 | Continued | NA |
| **4** | Cerebral infarction | Cilostazol | –66 | –9 | 5 | Continued | NA |
| **5** | Preoperative DIC treatment | Thrombomodulin | –7 | –1 | 3 | -5 | Operation |
| **6** | Preoperative DIC treatment | Thrombomodulin | 26 | -2 | 2 | 34 | Operation |

Supplemental table 2
